# Pilot study on exercise-induced placental transcriptomic changes and oxidative stress reduction in gestational diabetes mellitus

**DOI:** 10.1038/s41598-025-28642-x

**Published:** 2025-12-29

**Authors:** Xuemei Li, Qimei Yang, Lin Lu, Xia Zhang, Lidan He, Rongjin Lin, Jianbo Wu

**Affiliations:** 1 Department of Obstetrics and Gynecology, The First Affiliated Hospital of Fujian Medical University, Chazhong, Fuzhou, 350000 Fujian China; 2 Department of Obstetrics, The First People’s Hospital of Yunnan, Xishan, Kunming, 650032 Yunnan China; 3 Nursing Department, The First Affiliated Hospital of Fujian Medical University, Chazhong, Fuzhou, 350000 Fujian China

**Keywords:** Gestational diabetes mellitus, Physical activity, Oxidative stress, Immune regulation, Placental transcriptomics, Immunology, Endocrinology

## Abstract

Gestational diabetes mellitus (GDM) is a common pregnancy complication associated with adverse maternal and neonatal outcomes, characterised by inflammation and oxidative stress. While exercise interventions have been shown to alleviate some of these issues, the underlying molecular mechanisms, particularly in the placenta, remain poorly understood. This study investigates the impact of exercise on maternal immune function, oxidative stress, placental gene expression, and neonatal outcomes in GDM pregnancies. This pilot study involved 12 pregnant women, six with GDM and six with normal pregnancies. Participants were divided into four groups: normal pregnancies with exercise (NCE) or without exercise (NC), and GDM pregnancies with exercise (GDME) or without exercise (GDM). The exercise intervention included stationary cycling for 16 weeks, three times a week. Placental tissue and maternal blood were collected post-delivery. Placental gene expression was analysed using RNA sequencing, and oxidative stress was measured in maternal blood. Neonatal birth weight was significantly lower in the GDME group compared to the GDM group. Exercise significantly reduced oxidative stress and improved immune function in the GDME group, approaching levels observed in the NC and NCE groups. Transcriptomic analysis of placental samples revealed upregulation of antioxidant genes (e.g., *GPX3, MTCO1P40*) and downregulation of pro-inflammatory genes (e.g., *CCL21*), body weight regulatory gene (*IGFBP1*), indicating enhanced immune and metabolic balance, and regulation of fetal birth weight. Exercise interventions in GDM pregnancies improve placental function by modulating immune response and oxidative stress, improving neonatal outcomes. These findings support the inclusion of exercise in GDM management to optimise maternal and fetal health.

## Introduction

The prevalence of gestational diabetes mellitus (GDM) has been steadily increasing, currently estimated at 17.5% in China and 13.9% globally, posing significant short- and long-term risks to both mothers and fetuses, including macrosomia, miscarriage, and postpartum hypoglycemia^1–3^. GDM is a metabolic disorder characterised by chronic low-grade inflammation, with the persistent release of inflammatory cytokines affecting maternal health and potentially exerting detrimental effects on fetal development through alterations in the placental microenvironment^4,5^. The placenta plays a pivotal role in immune regulation and oxidative stress at the maternal fetal interface, and its structure and function have received considerable attention in GDM research^6^. In particular, inflammation has been identified as a central factor in GDM pathology^7,8^. A study by Francisco et al. explored how an unfavourable pro-inflammatory environment influences cellular energy metabolism in human placental tissues from pregnancies complicated by GDM^9^. Maternal systemic inflammation may impair placental fatty acid $\beta$-oxidation, adversely impacting fetal health, as demonstrated by both human clinical studies and animal models of pregnancy-related metabolic disorders^9,10^. While pharmacological treatments can regulate blood glucose levels, they often fail to address the underlying metabolic imbalance and may lead to adverse effects^11^. Non-pharmacological interventions, particularly exercise therapy, have emerged as essential strategies for GDM management. Regular physical exercise has been shown to enhance mitochondrial biogenesis, improve oxidative capacity, increase insulin sensitivity and vascular function, and reduce systemic inflammation, primarily based on studies in non-pregnant human populations and rodent models^12–15^. Previous studies have used various types of exercise, including aerobic (e.g., walking, cycling), resistance, and combined training, with evidence derived from both pregnant and non-pregnant human populations as well as rodent models^16^. Our intervention focused on moderate-intensity cycling, consistent with established guidelines^17^. Moreover, exercise may help reduce the insulin dosage required by pregnant women undergoing insulin therapy^18,19^. A growing body of evidence suggests that exercise is a preventive measure and a potential therapeutic strategy, improving maternal and fetal health and reducing complications through epigenetic modifications, as shown in human pregnancy cohorts and animal models of gestational metabolic disorders^11,20^. While exercise interventions have been shown to enhance metabolic dysregulation, the specific mechanisms influencing placental transcriptomics, maternal immunity, and oxidative stress remain poorly understood. Investigating the changes in placental transcriptomics and peripheral blood profiles following exercise in GDM patients could provide critical insights into how exercise impacts maternal and fetal health.

In this study, we compared the effects of exercise interventions on the placental transcriptome and oxidative stress in the peripheral blood of pregnant women with GDM. Due to its exploratory nature and limited sample size, this study was designated as a pilot study. Given the novelty of integrating exercise interventions with transcriptomic and immunological analyses in GDM, this study aimed to establish the approach’s viability, assess the protocols’ reliability, and identify key molecular targets and pathways influenced by exercise.

## Methods

### Study participants

All participating pregnant women signed a written informed consent after receiving an explanation of the study’s goal. This study was conducted under the Declaration of Helsinki. The study protocol was approved by the First Affiliated Hospital of Fujian Medical University (MTCA, ECFAH of FMU[2023]393). This pilot study is a component of a more extensive research project titled “Construction and Application of a Glucose Management Model for Pregnant Women with Gestational Diabetes Based on a Cloud Platform”. It aims to ascertain feasibility and pinpoint molecular targets before commencing large - scale recruitment. The subgroup presented herein was chosen for the initial transcriptomic profiling. The clinical trial registration number is ChiCTR2300073621 on 17/07/2023. From August 2023 to August 2024, the study was conducted at the First Affiliated Hospital of Fujian Medical University, recruiting participants with GDM diagnosed through a 75-gram oral glucose tolerance test (OGTT) between weeks 24 and 28, with criteria including fasting blood glucose $\ge$ 5.1 mmol/L, 1-hour blood glucose $\ge$ 10.0 mmol/L, or $\ge$ 2-hour blood glucose 8.5 mmol/L^21^. Six pregnant women with GDM and six normal pregnant women participated in the study. Participants were assigned to either an exercise or a control group based on their participation in the intervention. The exercise group underwent stationary cycling training 3 times a week, while the control group maintained regular physical activity. Exercise interventions commenced immediately after GDM diagnosis (24-28 weeks of gestation) and continued for 16±2 weeks until delivery. Participants wore Huawei WATCH FIT3 trackers to monitor heart rate and estimate caloric expenditure throughout the intervention. Caloric expenditure data were used solely for tracking adherence to the exercise protocol and not included in the outcome analysis, as consumer-grade wearable devices are known to have an error margin of approximately ±10%^22^.

### Protocol, study design

The participants were assigned to four groups based on their exercise frequency during pregnancy: regular exercise (NCE, n=3), non-exercise (NC, n=3), GDM exercise (GDME, n=3), and GDM without exercise (GDM, n=3). Exclusion criteria included a history of type 1 or 2 diabetes, hyperthyroidism, Cushing’s syndrome, pancreatitis, and any other condition affecting glucose levels; pregnancy-related complications like preeclampsia; and severe heart, liver, or kidney diseases. Twelve placental samples were promptly collected from the umbilical cord’s base following cesarean or vaginal delivery, and data collected included maternal age, gestational week, neonatal weight, OGTT results, and pre-pregnancy body mass index (BMI). The characteristics of all participants are summarised in Table 1.

Exercise intervention program and evaluation indices: the GDME and NCE group performed moderate-intensity aerobic exercise using stationary bicycles, and the intervention lasted for more than 6 weeks. Stationary bicycle aerobic exercise was performed three to four times weekly for 50 to 60 minutes^23^. A sports medicine specialist provided structured instruction, and exercise intensity was controlled using a Borg scale (13-14 points) and heart rate monitoring equipment to ensure that the heart rate did not exceed 140 beats per minute or 50%-70% of the heart rate reserve. All participants were provided with standardised exercise logs and a wearable Huawei WATCH FIT3 to ensure consistency and adherence. Exercise frequency, duration, and caloric expenditure were recorded daily. Additionally, participants received weekly supervision and feedback via an online platform to ensure protocol fidelity. This structured exercise regimen was based on current guidelines for physical activity during pregnancy (ACOG and ACSM recommendations) and adapted to the specific needs of women with GDM, intending to optimise metabolic and immune outcomes while minimising risks^24^. Patients in both groups received personalised dietary guidance; diets were adjusted by a registered dietitian based on multiple individualised factors, including blood glucose levels, prepregnancy BMI, gestational weight gain, physical activity level, macronutrient requirements, and dietary preferences. Caloric intake was strictly controlled through daily standardised dietary diaries, weekly nutritional counselling, real-time monitoring via dietary apps, and dynamic adjustments based on glycemic control. Participants had to maintain a consistent caloric intake aligned with their personalised targets, which were calculated based on basal metabolic rate, gestational energy needs, and weight management goals, targeting Institute of Medicine (IOM) GWG guidelines^25^. Gestational weight gain was monitored weekly and compared to the IOM recommendations for weight gain based on prepregnancy BMI categories. All participants maintained weight gain within the IOM-recommended range: 11.5–16 kg for normal-weight women and 7–11.5 kg for overweight women. No participant exceeded the upper limit of the recommended range. Patients also participated in an online educational course to learn diabetes management, dietary records, and blood glucose monitoring skills, as well as weight management to ensure that weight gain in pregnancy was in line with the recommended standards.

### Peripheral blood Flow cytometry

Peripheral blood collected < 24h postpartum, and erythrocytes were lysed using erythrocyte lysis buffer and centrifugation to isolate leukocytes.

Cells were stained with JC-1 working solution (freshly prepared by diluting JC-1 stock and assay buffer) and incubated at 37$^\circ$C for 20 minutes. After incubation, cells were washed with pre-chilled 1×JC-1 Assay Buffer and analysed within 30 minutes using flow cytometry. Data were processed in FlowJo software, focusing on quadrant Q3, which indicates mitochondrial membrane potential depolarisation.

Reagent 1 working solution was prepared for reactive oxygen species (ROS) detection by diluting with serum-free culture medium to a final concentration of 10 $\mu$M (or optimised between 0.1–20 $\mu$M based on pre-experiments). Reagent 2 working solution was prepared for positive controls by diluting 10 mM TBHP to a final concentration of 50 $\mu$M in a serum-free medium. Positive control wells were treated with Reagent 2 for 2 hours. Cells were incubated with Reagent 1 at 37$^\circ$C in the dark for 30–60 minutes. Following incubation, cells were centrifuged at 1000g for 5–10 minutes, washed 2–3 times with serum-free medium to remove extracellular DCFH-DA, and resuspended for flow cytometry analysis. FlowJo software analysed ROS levels, comparing positive, negative, and experimental groups.

### Sample collection and preparation

Twelve placenta samples (GDM, GDME, NC, NCE) were selected for transcriptome analysis via high-throughput sequencing. Fresh placental maternal surfaces were harvested promptly post-delivery from pregnant women. The sampling strategy involved collecting tissue samples at the three vertices of an equilateral triangle, with the umbilical cord’s base as the central point. The distance from the centre ranged between 3.0 and 5.0 cm, and the incision depth was maintained between 0.5 and 1.0 cm, ensuring a 1.0x1.0x1.0 cm tissue sample. Placental sampling followed the STANDARD Placental Collection Protocol, minimising variability in site/depth (3–5 cm from umbilical base; 1 cm³ volume)^26^. Following saline washing, the samples were treated with RNA Stabilisation Liquid and rapidly frozen in liquid nitrogen. All samples were then stored at -80$^\circ$C for subsequent RNA extraction.

### RNA extraction and quality control

The total RNA extraction was performed using the TRIzol method (Invitrogen, USA), and RNA quality was assessed through an Agilent 2100 Bioanalyzer employing the RNA 6000 Nano Kit (Agilent, USA), which determined the concentration, RNA Integrity Number (RIN), and the 28S/18S ratio. Protein content was also verified using the same bioanalyzer. To ensure the library’s quality, a quantitative real-time PCR (qRT-PCR) was employed to determine the effective concentration accurately. Sequencing was carried out on the BGISEQ 500 platform (Shenzhen Huada Gene) utilising SBS technology, generating 100 base pair (bp) reads with a read length of 100 bp. To ensure the accuracy and reliability of downstream analyses, the initial low-quality raw reads, characterised by poor quality, splice contamination, and a high proportion of unknown bases (N), were first preprocessed by filtering out these contaminants. This process resulted in the extraction of valid reads, called clean reads. Fragment-per-kilobase-million (FPKM) calculations, using HTSeq v0.6, normalised gene expression levels, adjusting for sequencing depth and length.

### Differential gene expression analysis

The reference genomes and the annotation file were downloaded from the ENSEMBL database. Bowtie 2 v 2.2.3 was used for building the genome index, and Clean Data was then aligned to the reference genome using HISAT 2 v 2.1.0. Differential gene expression (DEGs) analysis between biological replicates was conducted using DESeq2 v1.6.3, which models read counts as a binomial distribution and employs the false discovery rate adjustment for *P*-value calculation. Genes with q 0.05 and 1 were considered differentially expressed. DESeq 2 v 1.6.3 was employed for biological replicates, accounting for gene-specific dispersion and shared expression patterns. The Integrative Genomics Viewer (IGV) was employed to visualise mapping results using heatmaps and scatter plots. Gene Ontology analysis was performed to annotate the biological processes, cellular components, and molecular functions of DEGs. The Kyoto Encyclopedia of Genes and Genomes also analysed gene functions and biological pathways.

### qRT-PCR to validate candidate genes

qRT-PCR validated fifteen candidate genes (*RORB, IGFBP-1, OMD, CHRDL1, EPYC, CCL21, NDP, PRL, FOXL2NB, AADAC, JCHAIN, FGG, IMPDH1P2, NOC2LP2, MTCO1P40*) in GDM and GDME groups using a QuantStudio 6 Flex System. Specific primers were designed for each gene using PRIMER 5.0 software (Supplemental Table 1) to ensure gene specificity.

### Statistical analysis

Data are presented as mean ± standard deviation (Mean ± SD). Statistical analyses were performed using GraphPad Prism 9.0. Group comparisons for continuous variables were analyzed using unpaired Student’s t-tests. Flow cytometry data and qRT-PCR validation results were evaluated with t-tests, with significance set at *P*< 0.05. Differential gene expression analysis in RNA-seq used DESeq 2 (*q*< 0.05 and $|\log _{2} \textrm{FC} |$ > 1).

## Results

### Patient characteristics

Table 1 shows the clinical data of the participants. In the validation set, there were no significant differences (*P* value > 0.05) in maternal age, pre-pregnancy body mass index, fasting plasma glucose, 1-hour plasma glucose, and 2-hour plasma glucose between women with GDME and GDM, NCE, and NC. However, neonatal weight was lighter in patients with GDME than in GDM, and the difference was statistically significant (3050.0 ± 312.2g vs 3855.5 ± 336.0g).

**Table 1:** Baseline characteristics of women with GDME and GDM, NCE and NC.

Variables	GDM (n$=$3)	GDME (n$=$3)	*P* (GDM)	NC (n$=$3)	NCE (n$=$3)	*P* (N)
Maternal age(years)	$28.7 \pm 2.5$	$32.0 \pm 3.6$	0.262	$29.4 \pm 3.5$	$27.4 \pm 5.5$	0.623
gestational age at delivery(week)	$39.2 \pm 1.2$	$39.5 \pm 1.5$	0.481	$40.2 \pm 2.5$	$40.1 \pm 7.5$	0.984
Gestational weight gain (kg)	$14.2 \pm 1.8$	$13.5 \pm 1.2$	0.605	$13.8 \pm 1.5$	$12.9 \pm 1.1$	0.449
Type of delivery, n(vaginal/cesarean)	2/1	3/0	–	3/0	3/0	–
Neonatal weight (g)	$3855.5 \pm 336.0$	$3050.0 \pm 312.2$	0.038[1]	$3555.7 \pm 436.0$	$3153.2 \pm 635.0$	0.417
Fasting plasma glucose(mmol/L)	$5.4 \pm 0.3$	$5.6 \pm 0.4$	0.527	$4.0 \pm 0.1$	$3.9 \pm 0.5$	0.9998
1-h plasma glucose(mmol/L)	$10.3 \pm 0.4$	$10.5 \pm 0.6$	0.656	$7.3 \pm 0.2$	$6.3 \pm 0.8$	0.104
2-h plasma glucose(mmol/L)	$8.8 \pm 0.5$	$9.3 \pm 0.6$	0.33	$6.8 \pm 0.7$	$7.8 \pm 0.5$	0.114
BMI(kg/m2)	$21 \pm 2.6$	$22 \pm 1.6$	0.601	$20 \pm 1.6$	$19 \pm 7.6$	0.834
HbA1c(%)	$6.16 \pm 0.37$	$5.77 \pm 0.48$	0.328			
GA(%)	$16.90 \pm 1.32$	$15.40 \pm 2.18$	0.366	–	–	–

Data are expressed as means ± standard deviation(Mean ± SD); BMI, prepregnancy body mass index; GA, glycated albumin (%); Between-group comparisons were performed using independent samples t-tests.[1]*P*< 0.05

### Flow cytometry

**Figure 1:**
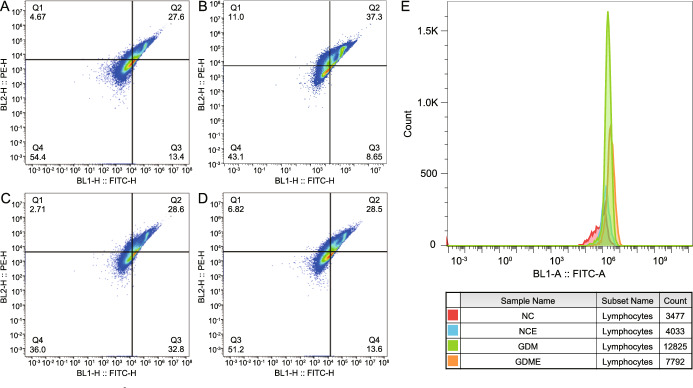
Representative flow cytometric analysis of mitochondrial membrane potential and ROS levels in maternal peripheral blood lymphocytes. Representative dot plots display the mitochondrial membrane potential in three groups: NC(**A**), NCE(**B**), GDM(**C**), and GDME(**D**). Each plot is divided into four quadrants (Q1, Q2, Q3, Q4) based on fluorescence intensity: Q1 (Upper Left): High BL2-H (PE) and low BL1-H (FITC), indicating early apoptotic cells with high mitochondrial activity. Q2 (Upper Right): High BL2-H (PE) and BL1-H (FITC), representing cells with high mitochondrial membrane potential. Q3 (Lower Right): Low BL2-H (PE) and high BL1-H (FITC), indicating cells with a collapsed mitochondrial membrane potential (key indicator of mitochondrial dysfunction). This population is particularly important in analysing oxidative stress and mitochondrial injury. Q4 (Lower Left): Low BL2-H (PE) and BL1-H (FITC), representing intact cells with no mitochondrial stress. Histogram of reactive oxygen species (ROS) levels in lymphocytes across the NC, NCE, GDM, and GDME groups(**E**). The x-axis represents the fluorescence intensity of BL1-A (FITC-A), indicating ROS production, while the y-axis represents the cell count. A clear rightward shift in the GDM group compared to the NC group demonstrates increased ROS levels, indicative of oxidative stress in GDM patients. The GDME group shows a partial reversal of ROS production toward normal levels, consistent with improved oxidative balance following intervention.

Flow cytometry revealed profound alterations in mitochondrial membrane potential and oxidative stress in maternal peripheral blood lymphocytes from women with GDM, modulated by exercise intervention. Supplemental Table 2 provides comprehensive quantitative data for all samples, while Fig. 1 presents representative flow cytometric profiles from individual subjects.

The percentage of JC-1 Q3-positive cells–a marker of mitochondrial depolarization–was markedly higher in the GDM group (34.20 ± 1.45%) than in the GDME group (15.33 ± 2.27%; P $=$ 0.0003; Fig. 1C and D; Supplemental Table 2), indicating that physical activity attenuates mitochondrial dysfunction in GDM. Exercise also reduced depolarization in normoglycemic pregnancies: the NCE group showed significantly lower Q3 values (7.90 ± 0.816%) compared to NC group (13.90 ± 0.56%; P $=$ 0.0005; Fig. 1A and B; Supplemental Table 2). In parallel, intracellular ROS levels were elevated in GDM (10,538.33 ± 2000.19 AU) versus NC (3,555.67 ± 69.70 AU) and were reduced by exercise in GDME (6,780.67 ± 893.20 AU; P $=$ 0.041; Fig. 1E; Supplemental Table 2). Together, these data show that GDM strongly disrupts mitochondrial and redox homeostasis, and that exercise significantly mitigates these effects–both in GDM and normoglycemic pregnancies.

### Overall RNA-Seq analysis and quality control

**Figure 2:**
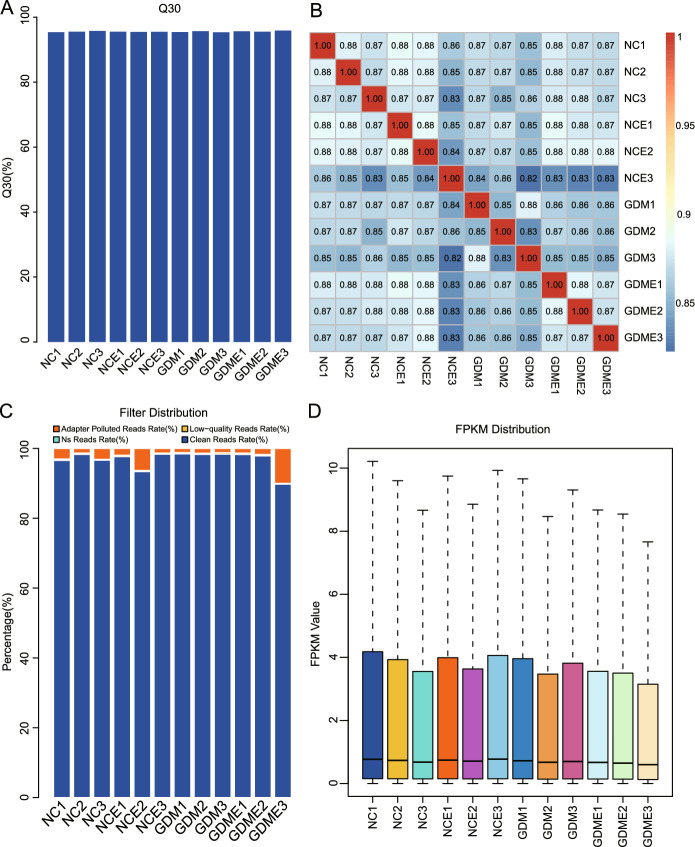
Overall RNA-Seq analysis and quality control. (**A**) Q30 quality control chart. (**B**) Sample correlation heat map. (**C**) Genetic Filtering Distribution Map. (**D**) Expression quantity box-style distribution chart. FPKM is used to estimate gene expression values quantitatively and can be corrected for read count for different sample sequencing depths and different gene lengths.

After excluding spliced and low-quality reads, the clean read samples were merged to analyse transcriptomic information. Q30 reflects the base quality level of sequencing, and the Q30 percentage of all samples in this project reached more than 95% (Fig. 2A). Samples within the same group exhibited high similarity in transcriptomic features, while the correlation between different groups was slightly lower, indicating notable transcriptomic differences. The correlation values for all samples in this study were generally high (above 80%), suggesting potential transcriptomic similarity; however, slight differences indicate differential expression between groups (Fig. 2B). This project included 12 samples, with the percentage of reads mapped to the reference genome exceeding 97% (Fig. 2C). The total number of clean reads surpassed 42,000,000, and the Q30 clean reads exceeded 95% (Supplemental Table 3), indicating the high quality of the obtained clean reads. Generally, the number of differentially expressed genes constitutes only a tiny fraction of the overall genes, suggesting that a limited number of differentially expressed genes have minimal impact on the distribution of sample expression levels. Figure2D presents a box plot of gene expression levels across all samples, revealing similar distribution patterns. Supplemental Table 3 summarises the main characteristics of each library, including clean reads, error rate, and GC content, as well as the rate of reads mapped to the reference genome and total gene expression content. The sample condition analysis confirms the data’s reliability and the correlation between samples.

### Analysis of differentially expressed genes (DEGs)

**Figure 3:**
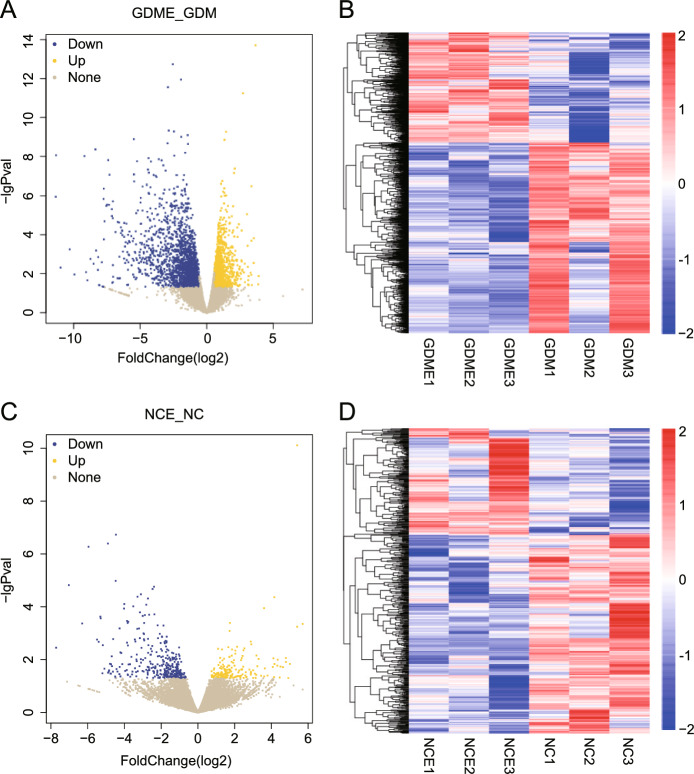
(**A**) and (**C**) Volcano plot of differentially expressed genes: The plot highlights the upregulated and downregulated genes between the experimental and control groups, with log2 fold change (x-axis) and statistical significance (-log10 p-value, y-axis). Genes with significant differential expression (adjusted *P*-value < 0.05) are colour-coded. (**B**) and (**D**) Heatmap of differentially expressed genes: The heatmap illustrates transcriptomic variations across 12 samples, showing distinct clustering of samples based on expression patterns. Samples within the same group exhibit consistent expression profiles, while intergroup differences align with the expected effects of the intervention. Genes included in the heatmap were filtered based on fold-change thresholds and statistical significance, ensuring the robustness of the clustering analysis. The red colour shows the number of upregulated genes, and the blue color shows the number of downregulated genes.

A volcano plot (Fig. 3A and C) and a class clustering heatmap (Fig. 3B and D) highlighted the substantial disparities between GDME and GDM, NCE and NC, suggesting distinct pathophysiological mechanisms.

### KEGG/GO enrichment analysis

**Figure 4:**
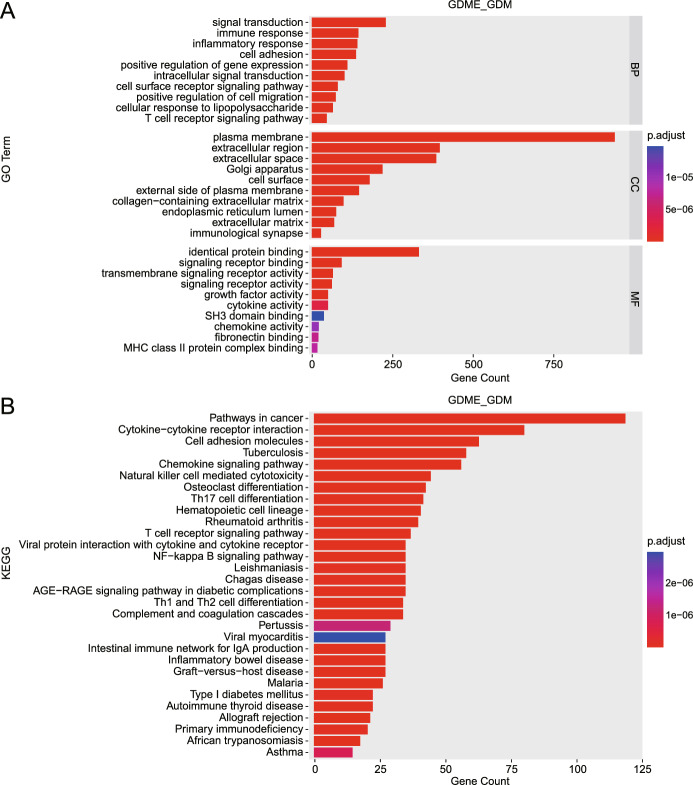
(**A**) GO enrichment analysis for DEGs identified in GDME and GDM groups. DEGs were categorised into three domains: biological process (BP), cellular component (CC), and molecular function (MF). (**B**) KEGG enrichment analysis for DEGs identified in GDME and GDM groups. The bar chart highlights the top enriched GO terms in each domain based on adjusted p-values (p.adjust).

For Gene Ontology (GO) analysis, these genes are categorised into three domains: biological process (BP), cellular component (CC), and molecular function (MF). We compiled a union set of enriched GO terms for GDME, GDM, NCE, and NC samples and plotted a distribution chart based on GO enrichment’s adjusted *P*-values (*P* adjust) (Supplemental Figure 1). The results of GO terms between GDME and GDM are shown in Supplemental Tables 4, 5 separately. Figure4A displays the top GO terms for GDME and GDM, each featuring the most abundant DEGs in the BP, CC, and MF categories. DEGs within the BP category were predominantly enriched in signal transduction, immune response, and inflammatory response processes. For the CC category, DEGs were significantly enriched in the cell periphery, Golgi apparatus, and endoplasmic reticulum. In the MF category, DEGs were mainly enriched in protein binding, signal receptor binding, transmembrane signal receptor activity, and growth factor activity.

Furthermore, we analysed the distribution of these transcripts in metabolic pathways using the KEGG database. We derived a union set of enriched pathways across all comparison groups and plotted a distribution chart based on the adjusted *P*-values of pathway enrichment (Supplemental Figure 2). Functional classification and pathway assignment were performed for GDM and GDME genes, and all DEGs were mapped to the KEGG database. The results revealed 130 significantly enriched pathways across three significant branches: genetic information processing, metabolism, and endocrine systems, with endocrine-related DEGs being the most prominent. This indicates that the regulatory response after exercise in GDM patients primarily focuses on endocrine mechanisms, such as thyroid, bone endocrine, and type 1 diabetes (Supplemental Figure 2). This provides crucial insights into understanding the regulatory mechanisms of exercise in GDM patients. Figure4B showcases the top 30 pathways with the most abundant DEGs in GDM and GDME, including the AGE-RAGE signalling pathway, energy metabolism, osteoclast differentiation, and the MAPK signalling pathway^27,28^. The genes related to these pathways are listed in Supplemental Table 6.

### Hub gene PPI network analysis

**Figure 5:**
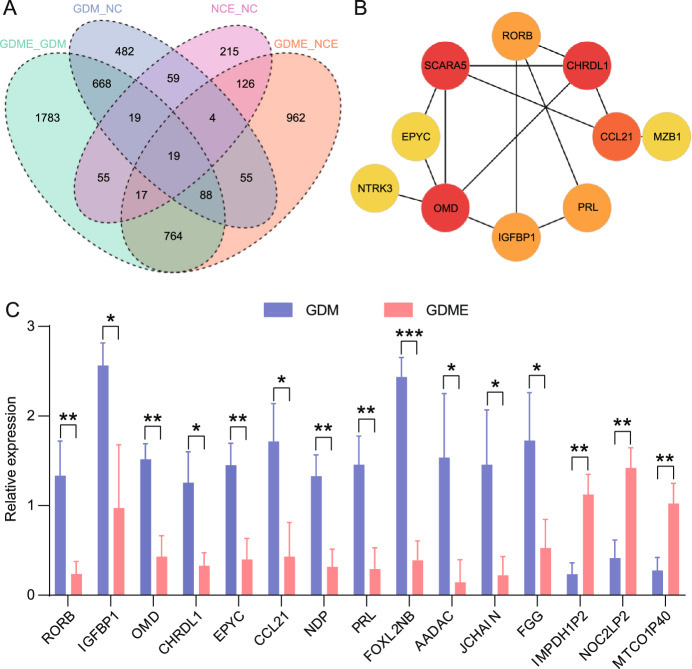
(**A**) Venn diagram showing overlapping differentially expressed genes between compared groups of the GDM, GDME, NC, and NCE. Each section represents the unique and shared genes across the analysed conditions. The overlapping regions indicate genes with consistent expression patterns across multiple groups, while the non-overlapping sections highlight genes uniquely influenced by exercise intervention or gestational diabetes. (**B**) The PPI network depicts interactions among DEGs identified in GDME and GDM groups. Nodes represent proteins encoded by DEGs, and edges indicate predicted or experimentally validated interactions. (**C**) qRT-PCR Validation of Differentially Expressed mRNAs. *P < 0.05. **P < 0.01. ***P < 0.001.

Microarray data revealed distinct gene expression patterns in placental tissues of GDME, GDM, NC, and NCE. This study employed DESeq2 to analyze differential gene expression, with the results of differentially expressed genes presented in Supplemental Table 7, 3,413 mRNAs exhibited differential expression, with 1,255 mRNAs upregulated and 2,158 mRNAs downregulated in GDME compared to GDM, meeting the criteria of a fold change (FC) $\ge$ 1.5 and a *P*-value < 0.05. The Venn diagram (Fig. 5A and Supplemental Table 8) reveals 54 overlapping DEGs between GDME/GDM and NCE/NC groups. Table 2 highlights gene expression differences between GDME and GDM (Table 2). Sequencing revealed 26 differentially expressed mRNAs in GDME, including 13 upregulated and 13 downregulated mRNAs. A PPI network, generated using STRING and Cytoscape, visualised the top ten downregulated genes (*OMD, CHRDL1, SCARA5, CCL21, RORB, NDP, IGFBP1, EPYC, PRL, and NTPK3 *). The degree algorithm identified key interaction genes (Fig. 5B).

**Table 2:** Differential gene expression in placental tissues from GDM and GDME groups.

Down regulation			Up regulation		
Gene name	$Log^2FC$	*P* value	Gene name	$Log^2FC$	*P* value
*CHRDL1*	−11.35	$1.13 \times 10^6$	*IMPDH1P2*	5.66	0.01
*IGFBP1*	−11.32	$8.73\times 10^9$	*NOC2LP2*	5.59	0.01
*EPYC*	−10.98	$4.94\times 10^3$	*MTCO1P40*	5.46	0.04
*CCL21*	−10.30	$5.67\times 10^4$	*VCX3B*	5.25	0.02
*NDP*	−9.94	0.01	*EPPIN*	5.00	$7.94\times 10^3$
*RORB*	−9.18	$9.59\times 10^9$	*KRT16P5*	4.73	0.02
*PRL*	−9.06	$8.38\times 10^3$	*CPNE4*	4.72	0.01
*FOXL2NB*	−8.96	0.02	*KNOP1P2*	4.48	0.04
*AADAC*	−8.73	$1.76\times 10^3$	*RN7SL708P*	4.47	0.03
*JCHAIN*	−8.49	$3.11\times 10^3$	*MED15P4*	4.34	0.04
*OMD*	−8.35	$4.37\times 10^9$	*H2AC21*	4.28	0.04
*IGLC2*	−8.31	$6.00\times 10^4$	*PRSS51*	4.07	0.02
*FGG*	−8.17	0.006	*GPX3*	4.25	0.03

### qRT-PCR validation

Fifteen differentially expressed mRNAs(*RORB, IGFBP1, OMD, CHRDL1, EPYC, CCL21, NDP, PRL, FOXL2NB, AADAC, JCHAIN, FGG, IMPDH1P2, NOC2LP2, MTCO1P40*) were validated using qRT-PCR. The results closely resembling those from mRNA sequencing (Fig. 5C and Supplemental Table 9) confirmed the accuracy of the sequencing data.

## Discussion

This study utilised high-throughput transcriptomic sequencing of placental tissue and peripheral blood flow cytometry to explore the effects of exercise intervention on pregnancy outcomes in patients with GDM. Compared with the non-intervention group, the exercise intervention group exhibited significantly lower neonatal birth weight, suggesting that exercise may modulate fetal growth toward a more normative trajectory. Moreover, transcriptomic analysis of the placenta revealed significant differential expression of key genes associated with immune regulation, oxidative stress, and regulation of fetal birth weight. In contrast, peripheral blood flow cytometric analysis confirmed mitochondrial membrane potential collapse and elevated oxidative stress in the placenta. Based on these findings, we hypothesise that exercise may improve immune inflammation and oxidative stress in GDM patients by modulating the expression of critical genes such as *CHRDL1, RORB, CCL21*, and *MTCO1P40*, and the regulation of fetal birth weight by the regulatory gene (*IGFBP1*).

### Mechanisms of exercise intervention in immune regulation

This study identified the potential role of exercise in mitigating GDM-related inflammation and reducing pathological complications by flow cytometry. We discovered that exercise intervention significantly downregulated the expression of *CCL21*. These results highlight the therapeutic potential of exercise in restoring oxidative homeostasis, and its incorporation into GDM management strategies may attenuate oxidative stress-related complications. *CCL21* is a pivotal pro-inflammatory cytokine that plays a role in T-cell homing and immune activation. The observed reduction implies that exercise might enhance immune dysregulation in GDM by inhibiting inflammatory signalling pathways, such as the NF-$\kappa$B pathway. This result aligns with those reported by Liu et al.^29^. Existing studies have shown that physical activity, including exercise by parents, can modulate inflammatory states in placental tissues. Several studies have shown that in pregnant women with GDM, placental tissues exhibit elevated expression of pro-inflammatory cytokines such as interleukin-6 (IL-6), TNF-$\alpha$, and chemokines including CCL2(C-C motif chemokine ligand 21) and TLR4, which are associated with increased macrophage infiltration and systemic immune dysregulation^30^. Interventions that reduce systemic inflammation may inhibit these inflammatory pathways. For instance, pharmacological treatments like metformin can alleviate excessive glucose intake-induced placental inflammation in GDM patients, which reduces pro-inflammatory cytokines and enhances placental defence mechanisms^31^. Exercise may exert similar effects. In a study involving pregnant women, maternal exercise has been shown to improve offspring metabolic health by increasing the expression of anti-inflammatory enzymes such as superoxide dismutase-3 (SOD3), thereby reducing oxidative pressure and inflammation in the placenta and promoting placental and fetal health^32^. These findings suggest that exercise exerts anti-inflammatory effects by modulating inflammatory pathways in the placenta and suppressing immune cell recruitment, which could be relevant for inflammatory regulation in GDM patients.

### Mechanisms of exercise intervention in alleviating oxidative stress

Additionally, exercise intervention notably upregulated the expression of antioxidant-related genes, including *GPX3* (glutathione peroxidase 3) and *MTCO1P40*. These gene expression alterations contribute to the restoration of redox balance in the placenta of GDM patients, thereby decreasing oxidative stress levels and ultimately providing protective effects on the health of both the mother and fetus. *GPX3*, a critical antioxidant enzyme, is essential for scavenging ROS and alleviating oxidative stress. A human cohort study reported that *GPX3* expression is often downregulated in placental tissues from GDM patients^29^, further corroborating our findings that exercise can effectively ameliorate oxidative stress in GDM pregnancies. Upregulation of genes such as *MTCO1P40* and *GPX3* reduces ROS in the placenta, thereby lowering oxidative stress, preventing oxidative damage, and maintaining cellular homeostasis^33^. Exercise promotes the expression of antioxidant enzymes, such as glutathione peroxidase (GPX) and superoxide dismutase (SOD), to restore placental redox balance^34^. Adaptive changes induced by exercise enhance mitochondrial antioxidant activity, which is critical for regulating oxidative stress in placental tissue. By improving redox homeostasis, exercise reduces placental oxidative damage and improves fetal health outcomes^35^. Although GDM placental tissues exhibit higher levels of oxidative stress markers, such as lipid peroxidation, exercise-induced antioxidant mechanisms can suppress these markers by enhancing mitochondrial adaptability and antioxidant enzyme expression^36^. This protective effect may be achieved by regulating mitochondrial dynamics, such as fusion and fission, to improve mitochondrial function and reduce oxidative stress markers like lipid peroxidation^37^. Moreover, exercise intervention was observed to suppress the expression of pro-oxidant genes such as *CHRDL1*, thus reducing the production of reactive oxygen species (ROS) and preserving normal placental function. In our study, flow cytometry results revealed that lymphocytes from women with GDM significantly reduced mitochondrial membrane potential, with 32.8% of cells in the Q3 quadrant and elevated ROS levels. These findings indicate the presence of substantial oxidative stress and mitochondrial dysfunction under GDM conditions. These results are consistent with previous studies. Francisco et al. reported that the chronic inflammatory state in GDM patients can impair mitochondrial biogenesis, affecting fatty acid $\beta$-oxidation and further exacerbating oxidative stress^9^. Similarly, Zhou et al. also observed a significant decrease in mitochondrial DNA copy number in peripheral blood mononuclear cells from GDM patients, suggesting that mitochondrial damage may be a key mechanism underlying GDM-related metabolic disturbances^38^. Our study further reveals that exercise intervention can significantly reverse this mitochondrial dysfunction. Importantly, our study demonstrates that exercise intervention can significantly reverse this mitochondrial dysfunction. In the GDME group, the proportion of cells in the Q3 quadrant decreased to 13.6%, approaching the level observed in the NC group (13.4%). These findings suggest that exercise has therapeutic potential in restoring mitochondrial homeostasis in GDM. These findings suggest that exercise has potential therapeutic value in restoring mitochondrial homeostasis. This result is consistent with the findings of Dempsey et al., who showed in a cohort of non-pregnant individuals that regular exercise training can enhance mitochondrial biogenesis, improve mitochondrial function, and reduce oxidative stress levels^39^.

### Interaction between immune regulation and oxidative stress

It is noteworthy that a positive feedback loop between inflammation and oxidative stress commonly exists in patients with GDM, which may exacerbate the progression of the disease^40^. Exercise intervention has effectively disrupted this vicious cycle, modulating the body’s immune and oxidative stress status. This study employed high-throughput RNA sequencing to systematically analyse gene expression profiles in placental tissues. GO functional enrichment analysis revealed that DEGs were predominantly enriched in biological processes such as immune regulation, redox reactions, and cellular metabolism. Furthermore, KEGG pathway analysis highlighted the significant involvement of the AGE-RAGE signalling, energy metabolism-related pathways, and the MAPK signalling pathway in the response to exercise intervention^27,28^. These findings suggest that exercise may improve placental function and optimise fetal and maternal health outcomes by modulating these key signalling pathways. Pro-inflammatory markers such as IL-6 and TNF-$\alpha$ are elevated in GDM patients and are known to increase ROS production. Conversely, ROS generated during oxidative stress can activate transcription factors such as NF-$\kappa$B, further promoting the production of pro-inflammatory cytokines and establishing a self-sustaining cycle^41^. Acute exercise can temporarily increase oxidative stress caused by muscle metabolism, leading to ROS production. Elevated IL-6 and TNF-$\alpha$ levels post-exercise confirm this response, which may exacerbate oxidative stress, particularly in metabolic disorders like GDM^42^. Although antioxidant defence mechanisms, such as glutathione peroxidase and SOD, are activated during exercise to counteract oxidative stress, their efficiency may be compromised in the pro-oxidative environment of GDM patients, potentially allowing inflammation and oxidative damage to persist^43^. Acute exercise may transiently exacerbate oxidative stress and inflammation in GDM patients due to temporary increases in ROS and inflammatory cytokines. This highlights the importance of adjusting exercise intensity and incorporating recovery strategies^44^. However, long-term, moderate-intensity exercise has been shown to enhance mitochondrial efficiency and increase antioxidant enzyme expression, thereby reducing oxidative stress and inflammation associated with GDM. Over time, such interventions can disrupt the positive feedback loop between inflammation and oxidative stress^45^. Therefore, in GDM patients, the interaction between inflammation and oxidative stress is particularly significant, especially post-exercise, when ROS and pro-inflammatory cytokines reinforce each other. While acute exercise may temporarily worsen this cycle, chronic, appropriately tailored exercise can mitigate these effects by enhancing antioxidant defences and improving mitochondrial function.

### Exercise affects fetal birth weight through placental function

Macrosomia and neonatal hypoglycemia are adverse pregnancy outcomes associated with GDM. In our study, neonatal birth weight in the GDME group was significantly lower than that in the GDM group ($3050.0 \pm 312.2$ g vs. $3855.5 \pm 336.0$ g), indicating that exercise intervention effectively controlled fetal weight gain and reduced the risk of macrosomia associated with GDM. Exercise is an effective methodology for preventing and managing GDM and has been shown to reduce the risk of these complications by modulating placental function^46,47^. Through placental transcriptomic analysis, several growth factors showed significant expression changes between the case and control groups, suggesting their potential involvement in regulating fetal weight by exercise. *CHRDL1*, which modulates the activity of bone morphogenetic proteins (BMPs), plays a key role in placental vascular development and fetal growth regulation^48^. Prolactin (PRL), a hormone with multiple functions in placental development and maternal metabolism^49^, was downregulated in the GDME group. Although PRL is associated with insulin resistance during pregnancy, its reduction in the exercise group may reflect improved metabolic balance and decreased inflammatory burden. Additionally, *IGFBP1*, a gene involved in fetal growth regulation, was downregulated, highlighting the role of exercise in fine-tuning growth factor signalling pathways^50^. *IGFBP-1* has been identified as a crucial factor influenced by exercise. IGFBP-1 is integral to glucose metabolism and fetal development, and its downregulation through physical activity may explain how exercise mitigates GDM severity. Prior research has demonstrated that aerobic and resistance exercise in non-pregnant individuals enhances the PI3K/Akt signalling pathway, leading to increased levels of IGF-1 and IGF-1 receptor^46^. Moreover, prolonged endurance exercise has elevated circulating IGFBP-1 concentrations in both human and rodent models, suggesting a conserved mechanism across species^51^. Notably, IGFBP-1 infusion can neutralise the insulin-like effects of IGF-1, thereby raising fasting blood glucose levels^52^. This indicates that IGFBP-1 may be critical in regulating blood glucose during and after exercise^53^. Based on our findings, it can be inferred that exercise may modulate the expression of fetal growth-related genes, such as *IGFBP1* and *PRL*, thereby influencing fetal birth weight and maternal adaptation to pregnancy, particularly under conditions of metabolic stress. The altered maternal environment observed in our study may impact key growth factor signalling pathways that directly regulate placental nutrient transport and fetal development, ultimately optimising the intrauterine growth environment and improving neonatal birth outcomes. These molecular alterations align with the observed clinical improvements in neonatal outcomes and maternal immune and metabolic profiles. This mechanism suggests a potential molecular pathway through which exercise benefits fetal birth weight by modulating placental function. Collectively, these findings provide a robust theoretical basis and molecular evidence supporting the implementation of exercise as an effective, non-pharmacological intervention strategy for pregnant women with GDM.

### Impact of gestational age at intervention initiation

In our study, the diagnosis of GDM was performed at 24–28 weeks of gestation, consistent with current clinical screening recommendations^54^. Consequently, our intervention began shortly after diagnosis, typically in the late second trimester. The timing of intervention initiation is important because both maternal metabolic adaptation and placental development are already well underway by this stage, which may influence the magnitude of metabolic and molecular responses to exercise. Previous exercise interventions in GDM vary in start time, though most studies initiate protocols within a similar gestational window (24–28 weeks) following standard diagnostic procedures^54^. A systematic review by Davenport et al. found that most randomised controlled trials on exercise in GDM commenced interventions between 24 and 30 weeks of gestation^55^. Huang et al. reported that moderate-intensity aerobic and resistance training in pregnant women with GDM, initiated at 25–26 weeks, improved maternal glycemic control and reduced insulin requirements, while earlier-start interventions have shown additional benefits on birth weight and maternal weight gain^55^. Given that placental development and immune regulation are dynamic processes, the timing of intervention may significantly influence the molecular changes observed in the placenta transcriptome^54^. In our study, the observed upregulation of antioxidant genes and downregulation of pro-inflammatory markers may be partly attributed to the timely initiation of exercise during a critical window of placental development.

### Limitations and future directions

We ensured that all participants adhered to the intervention protocol, with over 90% attendance for the required sessions, confirming the reliability of participant engagement in the study. This helped to mitigate potential confounding factors related to participant non-compliance. We also focused on the reliability of the experimental assays, particularly RNA-Seq. To ensure high-quality results, we implemented rigorous quality control measures during RNA sequencing, including obtaining over 42 million clean reads with Q30 scores above 95%. We also included detailed checks on sequencing depth and gene mapping rates, which validated the technical reliability of our data. Despite the meaningful results obtained, this study has several limitations. While transcriptomics and flow cytometry provided insights into the effects of exercise on placental immune and oxidative stress regulation, the specific functions and pathways of the identified genes require further investigation. Combining proteomics and single-cell transcriptomics in future studies could provide a more detailed understanding of the maternal-fetal interface. A limitation of this pilot study is the lack of baseline physical activity data, which could influence individual responses to the intervention. The small sample size and potential imbalance between groups may limit the generalizability of the findings. Future studies should involve larger cohorts and multi-centre collaborations to enhance external validity. This study focused on specific types and intensities of exercise but did not evaluate the differential effects of various exercise modalities, frequencies, and intensities. Future research should assess a broader range of exercise protocols to optimise intervention strategies for GDM patients. Recent studies have highlighted the influence of labour duration and oxytocin use on placental function and oxidative stress^56^. While our current study did not account for these factors, future research should consider their potential impact to refine the interpretation of molecular changes in placental tissues following maternal exercise interventions. Although this study indicated that the neonatal birth weight in the exercise intervention group was significantly lower than in the non-intervention group, changes in birth weight alone are insufficient to conclude an improvement in overall neonatal health outcomes. Clinically meaningful changes are typically defined based on deviations from the expected growth percentiles for gestational age (e.g., above the 90th percentile) or by extreme absolute values (e.g., below 2500 grams or above 4000 grams). Therefore, while the observed reduction in birth weight suggests that exercise may modulate fetal growth, further analysis is required to determine whether this change is beneficial. Clinically significant and to evaluate its actual impact on neonatal health outcomes. The current study primarily examined short-term effects during pregnancy. Longitudinal studies are needed to assess the long-term health benefits of exercise interventions for both mothers and offspring, providing more comprehensive data for clinical application.

## Conclusion

This study integrates placental transcriptomics and peripheral blood flow cytometry to demonstrate that exercise intervention improves immune-inflammatory and oxidative stress states in GDM patients. Exercise modulates the expression of key placental genes, such as *CHRDL1, RORB, CCL21, IGFBP1* and *MTCO1P40*, reducing neonatal birth weight and enhancing maternal and fetal health. These findings provide novel molecular mechanisms and theoretical support for non-pharmacological interventions in GDM management, highlighting the potential of exercise as a precision therapeutic strategy. Furthermore, this study lays the groundwork for future research into personalised GDM management and comprehensive intervention strategies.

## Supplementary Information

Supplementary Information.

## Data Availability

The datasets are available in the NCBI repository, PRJNA1240822.
