# Supplementary material for: Pilot study on exercise-induced placental transcriptomic changes and oxidative stress reduction in gestational diabetes mellitus
Source: Sci Rep. 2025 Dec 29;15:44821. doi: 10.1038/s41598-025-28642-x (PMC12749014; doi:10.1038/s41598-025-28642-x)
Supplement: Supplementary file 1 — Supplementary Information. [file 41598_2025_28642_MOESM1_ESM.zip › 9.29Supplementary material/Supplementary figure/Figure legend.docx]

**S1 Fig.** GO Enrichment Analysis of GDME with GDM, GDME with NCE, GDM with NC, NCE with NC

**S2 Fig.** KEGG Enrichment Analysis of GDME with GDM, GDME with NCE, GDM with NC, NCE with NC
