# Supplementary figures and images for: Pilot study on exercise-induced placental transcriptomic changes and oxidative stress reduction in gestational diabetes mellitus
Source: Sci Rep. 2025 Dec 29;15:44821. doi: 10.1038/s41598-025-28642-x (PMC12749014; doi:10.1038/s41598-025-28642-x)

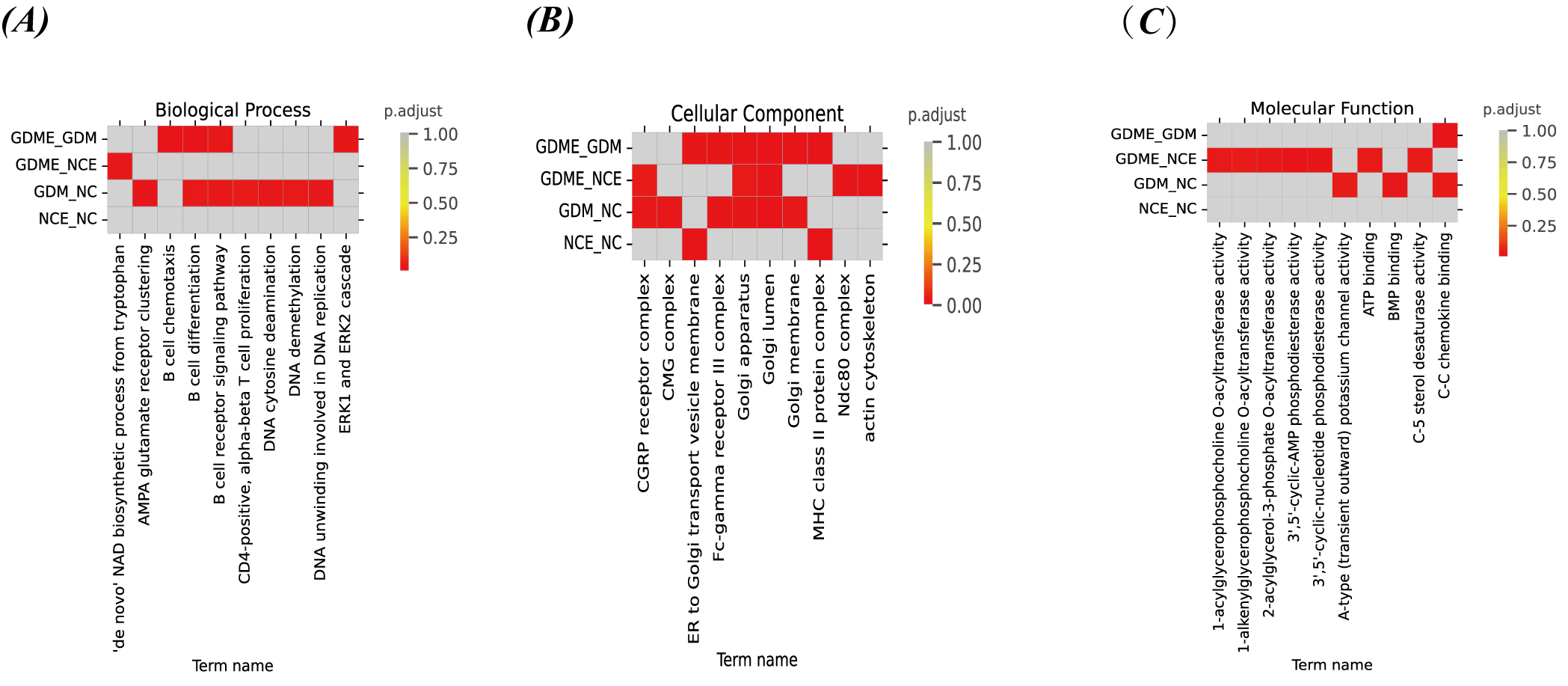

Supplement: Supplementary file 1 — Supplementary Information. [file 41598_2025_28642_MOESM1_ESM.zip › 9.29Supplementary material/Supplementary figure/S1 Fig.tif]

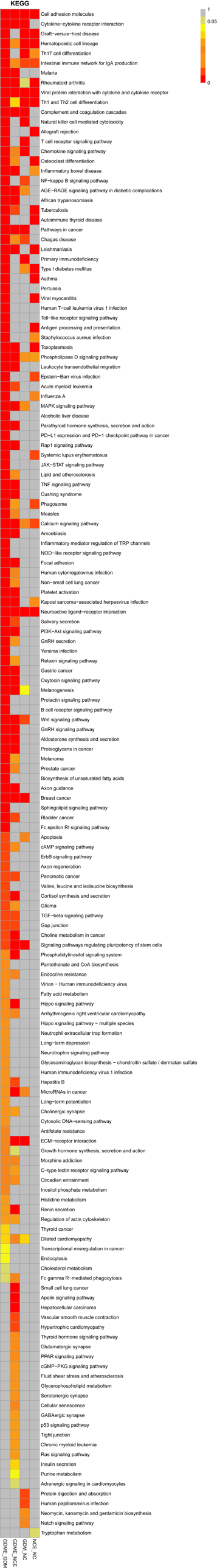

Supplement: Supplementary file 1 — Supplementary Information. [file 41598_2025_28642_MOESM1_ESM.zip › 9.29Supplementary material/Supplementary figure/S2 Fig.tif]
