# Supplementary material for: Pilot study on exercise-induced placental transcriptomic changes and oxidative stress reduction in gestational diabetes mellitus
Source: Sci Rep. 2025 Dec 29;15:44821. doi: 10.1038/s41598-025-28642-x (PMC12749014; doi:10.1038/s41598-025-28642-x)
Supplement: Supplementary file 1 — Supplementary Information. [file 41598_2025_28642_MOESM1_ESM.zip › 9.29Supplementary material/Supplementary Table.doc]

**S1 Table Primers for real time PCR**

| **Gene** | **Accession**  **Number** | **Primer Sequences** | **Production length**  **/bp** | **Tm/℃** |
| --- | --- | --- | --- | --- |
| RORB | NM_001365023.1 | Forward: AGTCTTCAGACCTCCCCAGT | 157 | 60 |
|  |  | Reverse: TCCCAGAGGACTTATCGCCA |  |  |
| IGFBP1 | XM_054358118.1 | Forward: CGCTGGGGCCATTACTCTTT | 115 | 60 |
|  |  | Reverse: ACGACCTCCTTTTCTCACCAC |  |  |
| OMD | NM_005014.3 | Forward: AGGCTGTGTCAGTGAATGCT | 107 | 60 |
|  |  | Reverse: GTTGCTGAATGTGCATCGGA |  |  |
| CHRDL1 | [NM_001143981.2](https://www.ncbi.nlm.nih.gov/entrez/viewer.fcgi?db=nucleotide&id=1653960919) | Forward: CAGAGTGGGTGAGAGATGGC | 184 | 60 |
|  |  | Reverse: GGGTAAGGAGTCTTCTGGGC |  |  |
| EPYC | [NM_004950.5](https://www.ncbi.nlm.nih.gov/entrez/viewer.fcgi?db=nucleotide&id=1519245222) | Forward: GGACTTGTTCTGGGACTTGTCA | 193 | 60 |
|  |  | Reverse:  GGAGCTCTCTGTTCCCTGAAG |  |  |
| CCL21 | [NM_002989.4](https://www.ncbi.nlm.nih.gov/entrez/viewer.fcgi?db=nucleotide&id=1519315496) | Forward: GCTGCTCCATCCCAGCTATC | 124 | 60 |
|  |  | Reverse:  TGTGGGGATGGTGTCTTGTC |  |  |
| NDP | [NM_000266.4](https://www.ncbi.nlm.nih.gov/entrez/viewer.fcgi?db=nucleotide&id=1519315601) | Forward: AGAAAGCTTCAGCCCGATCC | 177 | 60 |
|  |  | Reverse:  CACCAGCAGGGAGAGCATAG |  |  |
| PRL | [NM_001163558.3](https://www.ncbi.nlm.nih.gov/entrez/viewer.fcgi?db=nucleotide&id=1675158153) | Forward:  GGGGTTCATTACCAAGGCCA | 200 | 60 |
|  |  | Reverse:TGGATAGGATAGCCTCCGGG |  |  |
| FOXL2NB | [NM_001040061.3](https://www.ncbi.nlm.nih.gov/entrez/viewer.fcgi?db=nucleotide&id=1519473623) | Forward:  GAAATCTGCCGGTACTCGCT | 151 | 60 |
|  |  | Reverse:  GGGCTGGGGATTCTGAAAGC |  |  |
| AADAC | [XM_005247104.5](https://www.ncbi.nlm.nih.gov/entrez/viewer.fcgi?db=nucleotide&id=2217341752) | Forward:  ACAAAATTTGGTTGTCGGGAGC | 188 | 60 |
|  |  | Reverse:  TTAGAGCAGCACTTCCCACG |  |  |
| JCHAIN | [XM_054349955.1](https://www.ncbi.nlm.nih.gov/entrez/viewer.fcgi?db=nucleotide&id=2462597060) | Forward:  GGGAGTCCTGGCGGTTTTTA | 104 | 60 |
|  |  | Reverse:  TGGAAGTAATCCGGGCACAC |  |  |
| FGG | [NM_000509.6](https://www.ncbi.nlm.nih.gov/entrez/viewer.fcgi?db=nucleotide&id=1890333751) | Forward:  TTGGGCCACTTGGAAAACCC | 127 | 60 |
|  |  | Reverse:  GTCTCCAGCCTGTTTGGCT |  |  |
| IMPDH1P2 | NG_005150.7 | Forward:  GCTGCGTTAGAGGCGGGT | 114 | 60 |
|  |  | Reverse:  CCCATCCTCGGGCTGGTA |  |  |
| NOC2LP2 | NR_002826.2 | Forward:  GCATGAACGGCCAAACCTAC | 183 | 60 |
|  |  | Reverse:  TGTGGATGGACAAGGTCTGC |  |  |
| MTCO1P40 | NG_043281.1 | Forward:TAGGAATGGTGTGAGCCGTG | 161 | 60 |
|  |  | Reverse:GCCAGTTGGCTGAACCCTAA |  |  |

**S2 Table.** Flow Cytometry Analysis of Oxidative Stress and Mitochondrial Membrane Potential in Maternal Peripheral Blood Lymphocytes

| **Sample** | **NC1** | **NC2** | **NC3** | **NCE1** | **NCE2** | **NCE3** | **GDM1** | **GDM2** | **GDM3** | **GDME1** | **GDME2** | **GDME3** |
| --- | --- | --- | --- | --- | --- | --- | --- | --- | --- | --- | --- | --- |
| ROS Level(AU) | 3477 | 3610 | 3580 | 4033 | 3520 | 3960 | 12825 | 9120 | 9670 | 7792 | 6100 | 6450 |
| Mean ± SD | 3555.67 ± 69.70 | | | 3837.67 ± 277.03 | | | 10538.33 ± 2000.19 | | | 6780.67 ± 893.20 | | |
| *P* | 0.16 | | | | | | 0.041* | | | | | |
| Percent of JC-1 Q3(%) | 13.4 | 14.5 | 13.8 | 8.65 | 8.01 | 7.03 | 32.8 | 35.7 | 34.1 | 13.6 | 14.5 | 17.9 |
| Mean ± SD | 13.90 ± 0.56 | | | 7.90 ± 0.816 | | | 34.20 ± 1.45 | | | 15.33 ± 2.27 | | |
| *P* | 0.0005** | | | | | | 0.0003** | | | | | |

Data are shown as individual values and mean ± SD. P-values were calculated using unpaired Student’s t-test. **P*<0.05, ***P*<0.01.

**S3 Table The overall quality control of the RNA-Seq results**

| **Sample** | **NC1** | **NC2** | **NC3** | **NCE1** | **NCE2** | **NCE3** | **GDM1** | **GDM2** | **GDM3** | **GDME1** | **GDME2** | **GDME3** |
| --- | --- | --- | --- | --- | --- | --- | --- | --- | --- | --- | --- | --- |
| Raw Reads Number | 48068150 | 45266402 | 44494040 | 45688334 | 46071924 | 44958780 | 48505498 | 48273566 | 48788852 | 47552350 | 47532748 | 53110438 |
| Raw Bases Number | 7210222500 | 6789960300 | 6674106000 | 6853250100 | 6910788600 | 6743817000 | 7275824700 | 7241034900 | 7318327800 | 7132852500 | 7129912200 | 7966565700 |
| Clean Reads Number | 46429154 | 44498478 | 42999704 | 44628254 | 43037126 | 44224318 | 47762142 | 47457272 | 47986500 | 46748170 | 46536012 | 47686400 |
| Clean Reads Rate(%) | 96.59 | 98.3 | 96.64 | 97.68 | 93.41 | 98.37 | 98.47 | 98.31 | 98.36 | 98.31 | 97.9 | 89.79 |
| Clean Bases Number | 6964373100 | 6674771700 | 6449955600 | 6694238100 | 6455568900 | 6633647700 | 7164321300 | 7118590800 | 7197975000 | 7012225500 | 6980401800 | 7152960000 |
| Low-quality Reads Number | 199416 | 177806 | 109502 | 166938 | 132964 | 144662 | 164114 | 156024 | 205560 | 176148 | 182928 | 114854 |
| Low-quality Reads Rate(%) | 0.41 | 0.39 | 0.25 | 0.37 | 0.29 | 0.32 | 0.34 | 0.32 | 0.42 | 0.37 | 0.38 | 0.22 |
| Ns Reads Number | 0 | 0 | 0 | 0 | 0 | 0 | 0 | 0 | 0 | 0 | 0 | 0 |
| Ns Reads Rate(%) | 0 | 0 | 0 | 0 | 0 | 0 | 0 | 0 | 0 | 0 | 0 | 0 |
| Adapter Polluted Reads Number | 1439580 | 590118 | 1384834 | 893142 | 2901834 | 589800 | 579242 | 660270 | 596792 | 628032 | 813808 | 5309184 |
| Adapter Polluted Reads Rate(%) | 2.99 | 1.3 | 3.11 | 1.95 | 6.3 | 1.31 | 1.19 | 1.37 | 1.22 | 1.32 | 1.71 | 10 |
| Raw Q30 Bases Rate(%) | 94.74 | 95.16 | 95.48 | 95.07 | 94.45 | 95.22 | 95.09 | 95.4 | 94.98 | 95.33 | 95.16 | 94.54 |
| Clean Q30 Bases Rate(%) | 95.36 | 95.52 | 95.75 | 95.51 | 95.44 | 95.48 | 95.4 | 95.66 | 95.32 | 95.64 | 95.53 | 95.87 |
| Raw GC percent(%) | 52.82 | 51.87 | 55.49 | 52.73 | 53.98 | 47.27 | 52.16 | 48.95 | 52.65 | 53.34 | 53.7 | 55.25 |
| Clean GC percent(%) | 52.44 | 51.75 | 55.49 | 52.45 | 53.35 | 47.18 | 52.06 | 48.86 | 52.57 | 53.27 | 53.62 | 54.04 |

**S4 Table GO analysis of differentially expressed RNAs in GDM and GDME**

| **Ontology** | **Term name** | **Up Count** | **Up**  **Percent** | **Down**  **Count** | **Down**  **Percent** |
| --- | --- | --- | --- | --- | --- |
| **Molecular function** |  |  |  |  |  |
|  | transporter activity | 84 | 6.69 | 119 | 5.51 |
|  | translation regulator activity | 7 | 0.56 | 8 | 0.37 |
|  | transcription regulator activity | 90 | 7.17 | 170 | 7.88 |
|  | structural molecule activity | 40 | 3.19 | 57 | 2.64 |
|  | small molecule sensor activity | 2 | 0.16 | 2 | 0.09 |
|  | protein tag | 0 | 0 | 1 | 0.05 |
|  | protein folding chaperone | 3 | 0.24 | 9 | 0.42 |
|  | protein-containing complex destabilizing activity | 0 | 0 | 1 | 0.05 |
|  | molecular transducer activity | 51 | 4.06 | 230 | 10.66 |
|  | molecular sequestering activity | 1 | 0.08 | 4 | 0.19 |
|  | molecular function regulator activity | 129 | 10.28 | 259 | 12 |
|  | molecular carrier activity | 1 | 0.08 | 6 | 0.28 |
|  | molecular adaptor activity | 22 | 1.75 | 56 | 2.59 |
|  | general transcription initiation factor activity | 1 | 0.08 | 3 | 0.14 |
|  | cytoskeletal motor activity | 6 | 0.48 | 11 | 0.51 |
|  | catalytic activity | 329 | 26.22 | 636 | 29.47 |
|  | cargo receptor activity | 12 | 0.96 | 22 | 1.02 |
|  | binding | 743 | 59.2 | 1482 | 68.67 |
|  | ATP-dependent activity | 27 | 2.15 | 43 | 1.99 |
|  | antioxidant activity | 3 | 0.24 | 8 | 0.37 |
| **Cellular component** |  |  |  |  |  |
|  | synapse | 36 | 2.87 | 104 | 4.82 |
|  | supramolecular complex | 62 | 4.94 | 102 | 4.73 |
|  | protein-containing complex | 315 | 25.1 | 575 | 26.65 |
|  | other organism part | 0 | 0 | 3 | 0.14 |
|  | organelle | 650 | 51.79 | 1187 | 55 |
|  | nucleoid | 6 | 0.48 | 1 | 0.05 |
|  | membrane-enclosed lumen | 77 | 6.14 | 158 | 7.32 |
|  | membrane | 554 | 44.14 | 1103 | 51.11 |
|  | extracellular region | 91 | 7.25 | 307 | 14.23 |
|  | cellular anatomical entity | 1010 | 80.48 | 1926 | 89.25 |
|  | cell junction | 123 | 9.8 | 212 | 9.82 |
| **Biological process** |  |  |  |  |  |
|  | viral process | 11 | 0.88 | 12 | 0.56 |
|  | signaling | 43 | 3.43 | 98 | 4.54 |
|  | rhythmic process | 11 | 0.88 | 33 | 1.53 |
|  | response to stimulus | 284 | 22.63 | 809 | 37.49 |
|  | reproductive process | 73 | 5.82 | 155 | 7.18 |
|  | reproduction | 1 | 0.08 | 1 | 0.05 |
|  | pigmentation | 2 | 0.16 | 4 | 0.19 |
|  | multicellular organismal process | 175 | 13.94 | 383 | 17.75 |
|  | metabolic process | 468 | 37.29 | 799 | 37.03 |
|  | locomotion | 56 | 4.46 | 199 | 9.22 |
|  | localization | 268 | 21.35 | 451 | 20.9 |
|  | immune system process | 90 | 7.17 | 485 | 22.47 |
|  | homeostatic process | 106 | 8.45 | 205 | 9.5 |
|  | growth | 17 | 1.35 | 36 | 1.67 |
|  | developmental process | 315 | 25.1 | 644 | 29.84 |
|  | detoxification | 1 | 0.08 | 4 | 0.19 |
|  | cellular process | 813 | 64.78 | 1629 | 75.49 |
|  | cellular component organization or biogenesis | 328 | 26.14 | 557 | 25.81 |
|  | cell population proliferation | 31 | 2.47 | 134 | 6.21 |
|  | cell killing | 2 | 0.16 | 37 | 1.71 |
|  | cell aggregation | 5 | 0.4 | 3 | 0.14 |
|  | biological regulation | 666 | 53.07 | 1449 | 67.15 |
|  | biological process involved in intraspecies interaction between organisms | 2 | 0.16 | 7 | 0.32 |
|  | biological process involved in interspecies interaction between organisms | 60 | 4.78 | 266 | 12.33 |
|  | behavior | 25 | 1.99 | 68 | 3.15 |

**S5 Table GO analysis of differentially expressed RNAs in NC and NCE**

| **Ontology** | **Term name** | **Up Count** | **Up Percent** | **Down Count** | **Down Percent** |
| --- | --- | --- | --- | --- | --- |
| **Molecular function** |  |  |  |  |  |
|  | transporter activity | 17 | 9.39 | 16 | 4.8 |
|  | translation regulator activity | 1 | 0.55 | 1 | 0.3 |
|  | transcription regulator activity | 14 | 7.73 | 12 | 3.6 |
|  | structural molecule activity | 5 | 2.76 | 15 | 4.5 |
|  | protein tag | 0 | 0 | 1 | 0.3 |
|  | protein folding chaperone | 1 | 0.55 | 1 | 0.3 |
|  | molecular transducer activity | 14 | 7.73 | 40 | 12.01 |
|  | molecular function regulator activity | 8 | 4.42 | 40 | 12.01 |
|  | molecular carrier activity | 0 | 0 | 2 | 0.6 |
|  | molecular adaptor activity | 1 | 0.55 | 4 | 1.2 |
|  | general transcription initiation factor activity | 1 | 0.55 | 0 | 0 |
|  | cytoskeletal motor activity | 1 | 0.55 | 2 | 0.6 |
|  | catalytic activity | 45 | 24.86 | 89 | 26.73 |
|  | cargo receptor activity | 1 | 0.55 | 1 | 0.3 |
|  | binding | 92 | 50.83 | 201 | 60.36 |
|  | ATP-dependent activity | 4 | 2.21 | 4 | 1.2 |
|  | antioxidant activity | 0 | 0 | 1 | 0.3 |
| **Cellular component** |  |  |  |  |  |
|  | virion component | 1 | 0.55 | 0 | 0 |
|  | synapse | 10 | 5.52 | 11 | 3.3 |
|  | supramolecular complex | 5 | 2.76 | 10 | 3 |
|  | protein-containing complex | 40 | 22.1 | 62 | 18.62 |
|  | other organism part | 1 | 0.55 | 0 | 0 |
|  | organelle | 67 | 37.02 | 149 | 44.74 |
|  | membrane-enclosed lumen | 6 | 3.31 | 25 | 7.51 |
|  | membrane | 71 | 39.23 | 153 | 45.95 |
|  | extracellular region | 13 | 7.18 | 53 | 15.92 |
|  | cellular anatomical entity | 116 | 64.09 | 268 | 80.48 |
|  | cell junction | 17 | 9.39 | 25 | 7.51 |
| **Biological process** |  |  |  |  |  |
|  | viral process | 1 | 0.55 | 2 | 0.6 |
|  | signaling | 6 | 3.31 | 12 | 3.6 |
|  | rhythmic process | 4 | 2.21 | 4 | 1.2 |
|  | response to stimulus | 32 | 17.68 | 101 | 30.33 |
|  | reproductive process | 11 | 6.08 | 15 | 4.5 |
|  | multicellular organismal process | 29 | 16.02 | 55 | 16.52 |
|  | metabolic process | 57 | 31.49 | 114 | 34.23 |
|  | locomotion | 13 | 7.18 | 17 | 5.11 |
|  | localization | 36 | 19.89 | 51 | 15.32 |
|  | immune system process | 6 | 3.31 | 56 | 16.82 |
|  | homeostatic process | 13 | 7.18 | 38 | 11.41 |
|  | growth | 3 | 1.66 | 3 | 0.9 |
|  | developmental process | 36 | 19.89 | 83 | 24.92 |
|  | detoxification | 0 | 0 | 2 | 0.6 |
|  | cellular process | 100 | 55.25 | 221 | 66.37 |
|  | cellular component organization or biogenesis | 41 | 22.65 | 67 | 20.12 |
|  | cell population proliferation | 8 | 4.42 | 8 | 2.4 |
|  | cell killing | 1 | 0.55 | 5 | 1.5 |
|  | biological regulation | 75 | 41.44 | 194 | 58.26 |
|  | biological process involved in intraspecies interaction between organisms | 2 | 1.1 | 1 | 0.3 |
|  | biological process involved in interspecies interaction between organisms | 7 | 3.87 | 38 | 11.41 |
|  | behavior | 7 | 3.87 | 13 | 3.9 |

**S6 Table The top 30 pathways with the most abundant DEGs in GDM and GDME**

| **ID** | **Description** | ***P* value** |
| --- | --- | --- |
| map04514 | Cell adhesion molecules | 9.92E-26 |
| map04060 | Cytokine-cytokine receptor interaction | 1.74E-19 |
| map05332 | Graft-versus-host disease | 2.26E-16 |
| map04640 | Hematopoietic cell lineage | 8.06E-16 |
| map04659 | Th17 cell differentiation | 2.94E-15 |
| map04672 | Intestinal immune network for IgA production | 2.53E-14 |
| map05144 | Malaria | 3.35E-14 |
| map05323 | Rheumatoid arthritis | 2.27E-13 |
| map04061 | Viral protein interaction with cytokine and cytokine receptor | 2.66E-13 |
| map04658 | Th1 and Th2 cell differentiation | 3.27E-13 |
| map04610 | Complement and coagulation cascades | 5.98E-13 |
| map04650 | Natural killer cell mediated cytotoxicity | 9.69E-13 |
| map05330 | Allograft rejection | 1.31E-12 |
| map04660 | T cell receptor signaling pathway | 2.48E-12 |
| map04062 | Chemokine signaling pathway | 2.49E-12 |
| map04380 | Osteoclast differentiation | 4.49E-12 |
| map05321 | Inflammatory bowel disease | 5.05E-12 |
| map04064 | NF-kappa B signaling pathway | 2.54E-11 |
| map04933 | AGE-RAGE signaling pathway in diabetic complications | 1.60E-10 |
| map05143 | African trypanosomiasis | 3.31E-10 |
| map05152 | Tuberculosis | 3.73E-10 |
| map05320 | Autoimmune thyroid disease | 4.17E-10 |
| map05200 | Pathways in cancer | 5.60E-10 |
| map05142 | Chagas disease | 8.61E-10 |
| map05140 | Leishmaniasis | 3.34E-09 |
| map05340 | Primary immunodeficiency | 4.26E-09 |
| map04940 | Type I diabetes mellitus | 7.51E-09 |
| map05310 | Asthma | 5.62E-08 |
| map05133 | Pertussis | 9.10E-08 |
| map05416 | Viral myocarditis | 2.28E-07 |
| map05166 | Human T-cell leukemia virus 1 infection | 3.01E-07 |
| map04620 | Toll-like receptor signaling pathway | 3.67E-07 |
| map04612 | Antigen processing and presentation | 4.09E-07 |
| map05150 | Staphylococcus aureus infection | 5.54E-07 |
| map05145 | Toxoplasmosis | 8.21E-07 |
| map04072 | Phospholipase D signaling pathway | 1.06E-06 |
| map04670 | Leukocyte transendothelial migration | 1.07E-06 |
| map05169 | Epstein-Barr virus infection | 1.62E-06 |
| map05221 | Acute myeloid leukemia | 1.71E-06 |
| map05164 | Influenza A | 2.62E-06 |
| map04010 | MAPK signaling pathway | 3.46E-06 |
| map04936 | Alcoholic liver disease | 4.23E-06 |
| map04928 | Parathyroid hormone synthesis, secretion and action | 5.81E-06 |
| map05235 | PD-L1 expression and PD-1 checkpoint pathway in cancer | 6.96E-06 |
| map04015 | Rap1 signaling pathway | 7.78E-06 |
| map05322 | Systemic lupus erythematosus | 8.22E-06 |
| map04630 | JAK-STAT signaling pathway | 9.35E-06 |
| map05417 | Lipid and atherosclerosis | 9.84E-06 |
| map04668 | TNF signaling pathway | 1.96E-05 |
| map04934 | Cushing syndrome | 2.38E-05 |
| map04145 | Phagosome | 2.57E-05 |
| map05162 | Measles | 3.22E-05 |
| map04020 | Calcium signaling pathway | 3.54E-05 |
| map05146 | Amoebiasis | 3.69E-05 |
| map04750 | Inflammatory mediator regulation of TRP channels | 5.41E-05 |
| map04621 | NOD-like receptor signaling pathway | 5.82E-05 |
| map04510 | Focal adhesion | 6.08E-05 |
| map05163 | Human cytomegalovirus infection | 7.44E-05 |
| map05223 | Non-small cell lung cancer | 7.50E-05 |
| map04611 | Platelet activation | 8.78E-05 |
| map05167 | Kaposi sarcoma-associated herpesvirus infection | 0.000121718 |
| map04080 | Neuroactive ligand-receptor interaction | 0.000143671 |
| map04970 | Salivary secretion | 0.000169597 |
| map04151 | PI3K-Akt signaling pathway | 0.000172296 |
| map04929 | GnRH secretion | 0.000222219 |
| map05135 | Yersinia infection | 0.000233823 |
| map04926 | Relaxin signaling pathway | 0.000243053 |
| map05226 | Gastric cancer | 0.00027709 |
| map04921 | Oxytocin signaling pathway | 0.000304879 |
| map04916 | Melanogenesis | 0.000312997 |
| map04917 | Prolactin signaling pathway | 0.000361133 |
| map04662 | B cell receptor signaling pathway | 0.000366111 |
| map04310 | Wnt signaling pathway | 0.000388664 |
| map04912 | GnRH signaling pathway | 0.000423797 |
| map04925 | Aldosterone synthesis and secretion | 0.000423797 |
| map05205 | Proteoglycans in cancer | 0.000515069 |
| map05218 | Melanoma | 0.000537429 |
| map05215 | Prostate cancer | 0.00060984 |
| map01040 | Biosynthesis of unsaturated fatty acids | 0.000613679 |
| map04360 | Axon guidance | 0.000744402 |
| map05224 | Breast cancer | 0.000777374 |
| map04071 | Sphingolipid signaling pathway | 0.001015752 |
| map05219 | Bladder cancer | 0.001059117 |
| map04664 | Fc epsilon RI signaling pathway | 0.001172384 |
| map04210 | Apoptosis | 0.001438759 |
| map04024 | cAMP signaling pathway | 0.001668122 |
| map04012 | ErbB signaling pathway | 0.001688839 |
| map04361 | Axon regeneration | 0.001812012 |
| map05212 | Pancreatic cancer | 0.001812012 |
| map00290 | Valine, leucine and isoleucine biosynthesis | 0.001919993 |
| map04927 | Cortisol synthesis and secretion | 0.001925097 |
| map05214 | Glioma | 0.002206883 |
| map04350 | TGF-beta signaling pathway | 0.002220663 |
| map04540 | Gap junction | 0.002421045 |
| map05231 | Choline metabolism in cancer | 0.002643979 |
| map04550 | Signaling pathways regulating pluripotency of stem cells | 0.002785535 |
| map04070 | Phosphatidylinositol signaling system | 0.003437021 |
| map00770 | Pantothenate and CoA biosynthesis | 0.003497825 |
| map01522 | Endocrine resistance | 0.00375133 |
| map03260 | Virion - Human immunodeficiency virus | 0.004513861 |
| map01212 | Fatty acid metabolism | 0.004675986 |
| map04390 | Hippo signaling pathway | 0.004817796 |
| map05412 | Arrhythmogenic right ventricular cardiomyopathy | 0.004819262 |
| map04392 | Hippo signaling pathway - multiple species | 0.005201158 |
| map04613 | Neutrophil extracellular trap formation | 0.005210403 |
| map04730 | Long-term depression | 0.00523408 |
| map04722 | Neurotrophin signaling pathway | 0.005259671 |
| map00532 | Glycosaminoglycan biosynthesis - chondroitin sulfate / dermatan sulfate | 0.005470199 |
| map05170 | Human immunodeficiency virus 1 infection | 0.005925275 |
| map05161 | Hepatitis B | 0.006183862 |
| map05206 | MicroRNAs in cancer | 0.006800544 |
| map04720 | Long-term potentiation | 0.00690251 |
| map04725 | Cholinergic synapse | 0.007163874 |
| map04623 | Cytosolic DNA-sensing pathway | 0.007236482 |
| map01523 | Antifolate resistance | 0.007393229 |
| map04512 | ECM-receptor interaction | 0.007613156 |
| map04935 | Growth hormone synthesis, secretion and action | 0.008064914 |
| map05032 | Morphine addiction | 0.008312995 |
| map04625 | C-type lectin receptor signaling pathway | 0.008582757 |
| map04713 | Circadian entrainment | 0.008784097 |
| map00562 | Inositol phosphate metabolism | 0.009102558 |
| map00340 | Histidine metabolism | 0.009846541 |
| map04924 | Renin secretion | 0.009936504 |
| map04810 | Regulation of actin cytoskeleton | 0.011464877 |
| map05216 | Thyroid cancer | 0.011949061 |
| map05414 | Dilated cardiomyopathy | 0.013680745 |
| map05202 | Transcriptional misregulation in cancer | 0.015072615 |
| map04144 | Endocytosis | 0.015507371 |
| map04979 | Cholesterol metabolism | 0.017019468 |
| map04666 | Fc gamma R-mediated phagocytosis | 0.017131019 |

S7 Table Differentially expressed gene results in GDM and GDME, GDME and NCE, GDM and NC

| **Name** | **GDME_GDM** | **GDME_NCE** | **GDM_NC** |
| --- | --- | --- | --- |
| Up | 1255 | 662 | 939 |
| Down | 2158 | 1373 | 455 |
| Total | 3413 | 2035 | 1394 |

**S8 Table Overlap of differentially expressed genes found between GDME/GDM and NCE/NC populations**

| **ID** | **Symbol** | **NR:Evalue** |
| --- | --- | --- |
| ENSG00000198886 | Proton-antipo-M | 1.28E-180 |
| ENSG00000162747 | Ig-3 | 1.05E-178 |
| ENSG00000237541 | MHC-II-alpha | 2.40E-174 |
| ENSG00000204252 | MHC_II_alpha | 8.17E-173 |
| ENSG00000204287 | MHC_II_alpha | 1.03E-166 |
| ENSG00000254750 | Peptidase_C14 | 1.37E-160 |
| ENSG00000116132 | Homeobox | 3.71E-157 |
| ENSG00000103260 | Dendrin | 1.45E-150 |
| ENSG00000184557 | SH2 | 4.91E-150 |
| ENSG00000019582 | MHC2-interact | 2.18E-142 |
| ENSG00000049249 | TNFR_c6 | 4.50E-142 |
| ENSG00000112149 | V-set | 1.96E-139 |
| ENSG00000125740 | Atrophin-1 | 8.37E-137 |
| ENSG00000241399 | Lectin_C | 1.32E-132 |
| ENSG00000227039 | Atrophin-1 | 1.00E-130 |
| ENSG00000173372 | C1q | 1.37E-129 |
| ENSG00000153814 | zf-C2H2_aberr | 2.86E-127 |
| ENSG00000069424 | Aldo_ket_red | 5.56E-127 |
| ENSG00000102575 | Plasmodium_HRP | 2.44E-125 |
| ENSG00000186407 | V-set | 1.87E-120 |
| ENSG00000152766 | Ank_2 | 1.66E-118 |
| ENSG00000258227 | Lectin_C | 1.18E-113 |
| ENSG00000128709 | Homeobox | 2.14E-110 |
| ENSG00000169605 | BRICHOS | 3.59E-106 |
| ENSG00000168209 | RTP801_C | 1.15E-105 |
| ENSG00000090382 | Lys | 5.62E-100 |
| ENSG00000138685 | FGF | 7.05E-100 |
| ENSG00000108823 | Sarcoglycan_2 | 5.16E-99 |
| ENSG00000187608 | ubiquitin | 1.25E-97 |
| ENSG00000100097 | Gal-bind_lectin | 6.63E-92 |
| ENSG00000025708 | Glycos_transf_3 | 5.32E-89 |
| ENSG00000227154 | zf-RING_2 | 3.03E-88 |
| ENSG00000179344 | MHC_II_beta | 1.60E-82 |
| ENSG00000132329 | RAMP | 1.23E-80 |
| ENSG00000172346 | CSD | 1.60E-77 |
| ENSG00000175785 | PRIMA1 | 7.34E-76 |
| ENSG00000121570 | DCR | 3.64E-75 |
| ENSG00000090104 | RGS | 3.93E-71 |
| ENSG00000240065 | Proteasome | 8.63E-69 |
| ENSG00000142484 | L6_membrane | 2.17E-67 |
| ENSG00000165272 | MIP | 2.03E-65 |
| ENSG00000160221 | DUF1431 | 5.52E-62 |
| ENSG00000231389 | C1-set | 2.20E-57 |
| ENSG00000233523 | Rifin_STEVOR | 4.00E-47 |
| ENSG00000081041 | IL8 | 5.13E-43 |
| ENSG00000276085 | IL8 | 5.70E-40 |
| ENSG00000026751 | IL4 | 3.74E-36 |
| ENSG00000164825 | Defensin_beta | 9.49E-33 |
| ENSG00000276070 | IL8 | 7.69E-29 |
| ENSG00000198840 | Oxidored_q4 | 1.19E-19 |
| ENSG00000286467 | KBP_C | 7.92E-19 |
| ENSG00000235257 | Tmemb_161AB | 3.75E-16 |
| ENSG00000248809 | DUF2182 | 9.70E-08 |
| ENSG00000232320 | . | 4.37E-06 |

**S9 Table** **The expression levels of differentially expressed mRNAs**

| **Gene** | **GDM group (n=3)** | **GDME group (n=3)** | ***P*** |
| --- | --- | --- | --- |
| RORB | 1.333 ± 0.387 | 0.237± 0.140** | 0.0099 |
| IGFBP1 | 2.563 ± 0.254 | 0.974± 0.705 * | 0.0213 |
| OMD | 1.517 ± 0.174 | 0.432 ± 0.231 ** | 0.0029 |
| CHRDL1 | 1.256 ± 0.345 | 0.328 ± 0.145* | 0.0127 |
| EPYC | 1.452± 0.245 | 0.398± 0.236 ** | 0.0058 |
| CCL21 | 1.717± 0.421 | 0.431± 0.381* | 0.0172 |
| NDP | 1.328± 0.239 | 0.315±0.198** | 0.0048 |
| PRL | 1.457± 0.319 | 0.291± 0.237** | 0.0071 |
| FOXL2NB | 2.437±0.216 | 0.389±0.216*** | 0.0003 |
| AADAC | 1.536±0.714 | 0.145±0.251* | 0.0344 |
| JCHAIN | 1.456±0.612 | 0.221±0.211* | 0.0298 |
| FGG | 1.726±0.535 | 0.526±0.321* | 0.0291 |
| IMPDH1P2 | 0.234±0.126 | 1.123±0.225** | 0.004 |
| NOC2LP2 | 0.414±0.201 | 1.421±0.225** | 0.0044 |
| MTCO1P40 | 0.275±0.145 | 1.023±0.225** | 0.0084 |

GDM: gestational diabetes mellitus; GDME: gestational diabetes mellitus exercise group. Data are presented as mean ± standard deviation. Compared with the GDM group, **P*<0.05, ***P*<0.01.
